# GhSPX1s Interact with GhPHR1A and GhPHL1A in Regulating Phosphate Starvation Response in Cotton

**DOI:** 10.3390/biology14080916

**Published:** 2025-07-23

**Authors:** Nuerkaimaier Mulati, Miaomiao Hao, Yuxin Yang, Yanping Shi, Guanghui Xiao, Liping Zhu

**Affiliations:** 1 College of Life and Geographic Sciences, Kashi University, Kashi 844000, China; 18899355590@163.com; 2 Key Laboratory of Biological Resources and Ecology of Pamirs Plateau in Xinjiang Uygur Autonomous Region, Kashi University, Kashi 844000, China; 3 College of Life Sciences, Shaanxi Normal University, Xi’an 710119, China; mmhao@snnu.edu.cn (M.H.); 18877024122@163.com (Y.Y.); 4 Key Laboratory of Xinjiang Phytomedicine Resource and Utilization of Ministry of Education, College of Life Sciences, Shihezi University, Shihezi 832003, China; 15700977637@163.com; 5 National Key Laboratory of Cotton Bio-Breeding and Integrated Utilization, School of Life Science, Henan University, Kaifeng 475000, China; 13843021895xgh@163.com

**Keywords:** cotton, SPX, PHR, phosphorus signaling, protein interaction

## Abstract

SPX (*SYG1/Pho81/XPR1*) family genes play a pivotal role in phosphorus signaling, phosphorus uptake, and phosphorus translocation in plants. However, to date, the SPX family genes have not been systematically investigated in cotton. In this study, we conducted a genome-wide analysis and identified 44 SPX family genes in *Gossypium hirsutum*, classifying them into four subfamilies (SPX, SPX-MFS, SPX-EXS, and SPX-RING) based on conserved domains. An expression analysis revealed that the majority of SPX family genes were highly expressed in the root and stem. We identified hormone response, stress response, low-temperature response, and PHR1 binding sequence (P1BS) *cis*-elements in the promoters of the SPX genes. Additionally, the expression of *GhPHO1-4*, *GhSPX1-1/1-2/1-3*, and *GhSPX-MFS2-1/2-2* was significantly altered under phosphorus-deficient conditions and may be involved in the regulation of Pi response. A Y2H assay suggested that GhSPX1-1 interacts with GhPHR1A and GhSPX1-2 interacts with GhPHL1A. Our findings provide a basis for further cloning and functional verification of genes related to the regulatory network of low phosphorus tolerance in cotton.

## Simple Summary

This study investigated how cotton plants respond to phosphorus deficiency, a major challenge in agriculture that affects crop growth and yield. The researchers identified 44 genes in cotton belonging to the SPX family, which play a key role in phosphorus uptake and regulation. By analyzing these genes, the authors discovered that most genes are active in roots and stems, helping the plant adapt to low-phosphorus conditions. They also found that certain SPX proteins interact with other regulatory proteins, forming a system that fine-tunes phosphorus use in the plant. Using advanced computational tools, the team predicted how these proteins bind together, providing insights into their function. The findings could help develop cotton varieties that grow better in phosphorus-poor soils, reducing fertilizer use and improving sustainability in farming. This research offers a foundation for breeding more resilient crops, benefiting cotton farmers and the environment.

## 1. Introduction

Phosphorus is an essential component of plant organisms, participating in plant development through photosynthesis, respiration, membrane structures, and signal transduction pathways. Plants primarily absorb inorganic phosphorus in the form of phosphates; however, the majority of phosphorus in the soil exists as organic phosphorus and insoluble salts, which reduces the solubility and mobility of phosphorus, making it a major factor that limits plant growth [1,2]. Phosphorus starvation in plants refers to a metabolic stress response triggered when the soil available phosphorus (AP) concentration falls below 5 μM. Approximately 40–60% of terrestrial ecosystems experience phosphorus limitation. In field cotton cultivation, China’s major cotton-producing regions are concentrated in Xinjiang and similar areas. These regions primarily consist of saline–alkali soils, sandy beaches, and arid sandy lands. Most soils in these areas are calcareous, which exhibit strong phosphorus fixation capacity. This leads to severely insufficient AP content in the soil. In the course of long-term adaptation, plants have evolved a variety of strategies to enhance the absorption of phosphorus from the soil. For example, they maximize the contact area between the plant root system and the soil to absorb more phosphorus [3]. They also secrete organic acids through the roots, which reduce the soil pH and thereby activate phosphorus in the soil, promoting the utilization of insoluble phosphates by plants [4]. Additionally, plants form symbiotic relationships with arbuscular mycorrhizal fungi, enhancing the acquisition of phosphorus [5,6]. Furthermore, plants have evolved a complex signaling system to regulate the acquisition, utilization, and homeostasis of phosphorus. The *SPX* gene family and the phosphorus starvation response system play a central role in this process [7].

Members of the SPX gene family possess the SPX domain (Pfam: PF03105) and are named after the yeast suppressor of *gpa1* (*SYG1*), phosphate-regulated cyclin-dependent kinase inhibitor 81 (*PHO81*), and xenotropic and polytropic retrovirus receptor 1 (*XPR1*) [2,8]. The conserved SPX domain is typically located at the N-terminus, and based on the differences in the C-terminal domains, the SPX gene family is divided into four subfamilies: the SPX subfamily proteins that contain only the SPX domain; the SPX-EXS (*ERD1*, *XPR1*, and *SYG1*) subfamily, which includes both the SPX and EXS domains; the SPX-MFS (major facility super-family) subfamily, which contains the SPX and MFS domains; and the SPX-RING (really interesting new gene finger domain) subfamily, which includes the SPX and RING domains. Different subfamily members play distinct roles in phosphorus signaling, phosphorus uptake, and phosphorus translocation in plants [7,9].

In *Arabidopsis thaliana* and rice, there are four and six SPX proteins, respectively [2]. In eukaryotes, the signaling molecule inositol pyrophosphate (InsPs) within the cell can bind to the SPX domain, enabling SPX to sense the intracellular phosphorus content and subsequently regulate the uptake and translocation of phosphorus [10,11,12,13]. SPX proteins, through interactions with PHR1, inhibit the transcriptional regulation of downstream low-phosphorus response-related genes [14,15]. Under phosphorus-sufficient conditions, rice OsSPX1-6 can inhibit the binding of OsPHR2 to the P1BS sequence on the promoter of target genes by interacting with the C-terminus of OsPHR2, down-regulating the expression of phosphorus starvation response genes. Under low-phosphorus conditions, SPX proteins are degraded, which facilitates the release of OsPHR2 from the PHR2-SPX complex and up-regulates the expression of phosphorus starvation response genes [15,16,17,18]. AtSPX4 can interact with the transcription factor AtPAP1, and under phosphorus deficiency, AtPAP1 is released to activate the accumulation of anthocyanins [19]. Additionally, rice SPX-PHR also regulates the colonization induced by arbuscular mycorrhiza (AM) under phosphorus-deficient conditions [5].

There are 11 and 3 SPX-EXS proteins in *Arabidopsis thaliana* and rice, respectively [2]. Proteins containing both the SPX and EXS domains are known as the phosphate 1 (PHO1) family and respond to phosphate deficiency by mediating the transport of phosphorus from root to shoot [20]. In Arabidopsis, AtPHO1 and AtPHO1; H1 are widely involved in plant phosphorus homeostasis and can transport Pi from roots to shoots under phosphate deficiency [21]. OsPHO1;2, a homolog of AtPHO1, has also been shown to respond to phosphorus deficiency by mediating the transfer of phosphorus from roots to shoot [22]. OsPHO1;2 regulates phosphorus homeostasis in seeds, particularly in the endosperm cells [23]. It transports Pi out of the xylem in the vascular bundles and excess Pi from the endosperm cells and reloads it into the phloem of the diffuse vascular bundles, constituting Pi storage in seeds [24].

*Arabidopsis thaliana* and rice each contain three and four SPX-MFS proteins, respectively [2]. SPX-MFS, which is localized on the vacuolar membrane and responsible for phosphorus storage, is also named VPT (vacuolar phosphate transporter) [25]. *AtVPT1* mutation leads to the poor ability of plants to cope with changes in external phosphorus concentration [22]. There is an auto-inhibitory domain within the AtVPT1 protein; when the cytosolic Pi concentration is high, high concentrations of InsPs bind to the SPX domain of AtVPT1, activating its phosphate transport activity and transferring Pi into the vacuole; whereas under Pi deficiency, AtVPT1 inhibits its own phosphate transport function, thereby inhibiting the transport of Pi from the cytoplasm to the vacuole [26]. In the *vpt1/vpt3* double mutant, the phosphorus concentration in the roots and leaves decreases, but the phosphorus level in flowers increases with excessive phosphorus accumulation, leading to reduced fertility and phosphorus toxicity in the mutant [27]. OsSPX-MFS1 proteins maintain phosphorus homeostasis in plants by mediating Pi flow from the cytoplasm to the vacuole [28], while OsSPX-MFS3 is located in the tonoplast of rice protoplasts mediating Pi transport from the vacuole to the cytoplasm [29].

*Arabidopsis thaliana* and rice each have two SPX-RING proteins [2]. This subfamily belongs to the ubiquitin E3 ligases, and the SPX-RING proteins are named NLA (Nitrogen Limitation Adaptation) because they participate in Pi homeostasis in a NO3-dependent manner [30]. In rice, *OsNLA1* functions as an E3 ligase in the ubiquitination process, and by interacting with the phosphate transporter OsPT8, it targets OsPT8 for degradation via the 26S proteasome pathway, regulating the phosphorus balance of the plant [31]. AtNLA1 can ubiquitinate and degrade PHT1, and in the *nla* mutant, Pi uptake and intracellular Pi content increase, and miR827 can regulate *NLA*; under Pi starvation conditions, miR827 up-regulates and targets *NLA* mRNA to degrade *NLA* mRNA and inhibit the degradation of PHT1, thus activating Pi uptake and Pi transport from the roots to stems, and *AtNLA* negatively regulates Pi uptake [30,32].

To date, SPXs have been widely characterized in several plant species. There are 33 SPX genes in maize [33], 19 in *Solanum lycopersicum* [34], 20 in Tea-Oil Camellia [35], and 46 in wheat [36]. Cotton is an important natural fiber crop, which is widely cultivated in the world. Phosphorus deficiency will seriously affect the cotton yield, and the development of low-phosphorus tolerant cotton varieties is crucial for the sustainable development of the cotton industry. However, the identification of phosphorus-efficient genes in cotton is still very limited, and there have been no reports on the systematic analysis of the cotton SPX gene family and its molecular mechanisms involved in the low-phosphorus response. This study conducted a systematic analysis of the cotton SPX gene family, all putative SPX genes in *G. hirsutum* genome were identified, and their structural characteristics and conserved motifs were analyzed. The *cis*-acting elements of SPX family members were analyzed, and the expression levels of SPX family members were analyzed under different tissues and abiotic stress treatments. Moreover, the interaction of GhSPX1 with GhPHR1 and GhPHL1 in cotton was verified. This lays the foundation for the exploration of phosphorus-efficient genes in cotton and the breeding of cotton varieties that are tolerant to low-phosphorus conditions.

## 2. Materials and Methods

### 2.1. Genome-Wide Identification of the Cotton SPX Gene Family

The upland cotton *Gossypium hirsutum* ‘TM-1’ and Arabidopsis genome sequences were obtained from the CottonMD website (https://yanglab.hzau.edu.cn/CottonMD/download.1, accessed on 15 July 2025) and TAIR 10 (http://www.arabidopsis.org/, accessed on 15 July 2025), respectively. Other plant genome sequences used in this study were downloaded from Phytozome (https://phytozome.jgi.doe.gov/pz/portal.html, accessed on 15 July 2025). BLASTP-2.16.0 with default parameters was used to further identify the SPX proteins with 20 AtSPX sequences as the queries based on a homology search. After removing redundant sequences and incomplete ORF sequences, the NCBI Conserved Domain Search database (https://www.ncbi.nlm.nih.gov/Structure/bwrpsb/bwrpsb.cgi, accessed on 15 July 2025) and SMART7 tools (http://smart.embl-heidelberg.de/, accessed on 15 July 2025) were used to confirm the presence of characterized domains in the candidate sequences. The putative members of the SPX family and their gene sequences were identified and defined for further analyses. The number of amino acids and physicochemical parameters, including the molecular weight (kDa) and pI of each *GhSPX* protein, were calculated using ExPASy3.0 (https://web.expasy.org/compute_pi/, accessed on 15 July 2025). Subcellular location prediction was conducted using the CELLO v.2.5 (https://cello.life.nctu.edu.tw/, accessed on 15 July 2025) online server.

### 2.2. Phylogenetic Tree Analysis

To explore the evolutionary relationships among *Arabidopsis thaliana* and *Gossypium hirsutum*, we performed multiple alignments based on 20 AtSPXs and 44 GhSPXs using CLUSTALW2 [37], and the alignment results were employed to construct a phylogenetic tree using the neighbor joining method (NJ) in MEGA 11 with 1000 bootstrap replicates [38]. iTOL.v7 software (https://itol.embl.de/, accessed on 15 July 2025) was employed for visualization and modification of the phylogenetic tress [39].

### 2.3. Chromosome Location

The chromosomal location data of GhSPXs were extracted from the *Gossypium hirsutum* genome annotation file. This information was also used to construct chromosomal mapping with TBtools-II v2.322 [35].

### 2.4. Analyses of Gene Structure and Conserved Motifs

The CD-search tool (https://www.ncbi.nlm.nih.gov/Structure/bwrpsb/bwrpsb.cgi, accessed on 15 July 2025) was used to predict the conserved domains of cotton SPX genes translated into their corresponding protein sequences with default parameters. The gene structure of cotton SPX transcription factors was drawn using TBtools based on a *Gossypium hirsutum* genome annotation file.

### 2.5. Promoter Cis-Acting Element Analysis

To understand the possible regulation and response mechanism of cotton genes, the 2 kb upstream of genome sequences were obtained from each GhPHRs, and PlantCARE1 (http://bioinformatics.psb.ugent.be/webtools/plantcare/html/, accessed on 15 July 2025) was used to predict cis-acting elements of promoters [40] and finally visualized with TBtools-II v2.322 software.

### 2.6. Expression Pattern Analysis of GhSPXs and GhPHRs Using RNA-Seq Data

The tissue expression specificity of the *GhSPX* and *GhPHR* genes were analyzed by using RNA sequencing data of *Gossypium hirsutum* ‘TM-1’ [41] during different development stages and the downloaded stress treatments. The detailed cotton tissue samples come from the leaves, roots, and stems of 2-week-old *Gossypium hirsutum* ‘TM-1’plants; the anther, epicalyx, petal, pistil, sepal, and torus of whole mature flowers of *Gossypium hirsutum* ‘TM-1’plants; the cotton ovules from 0, 1, 3, 5, 10, and 20 day post anthesis; and the cotton fibers from 10, 20, and 25 day post anthesis. Libraries were sequenced at 2 × 100 bp on the Illumina HiSeq 2000 platform (Illumina, San Diego, CA, USA). To screen *GhSPX* and *GhPHR* genes in response to low phosphorus stress, the differential expression gene of low-phosphorus stress in *Gossypium hirsutum* were used [42]. The normalized expression values of the Fragments Per Kilobase of exon model per Million mapped fragments (FPKM) were z-score-transformed and clustered using Euclidean distance with complete linkage. Color gradients represented relative expression levels (red: up-regulated; blue: down-regulated). The heatmap was annotated with sample groups and key gene clusters. Visualization expression levels were optimized using the R-4.5.0 package pheatmap 1.0.12.

### 2.7. Yeast Two-Hybrid (Y2H) Assays

The matchmaker GAL4 two-hybrid system was used for yeast two-hybrid (Y2H) assays. Full-length *GhSPX1-1*, *GhSPX1-2* and *GhPHR1A*, *GhPHR1D*, *GhPHL1A*, and *GhPHL1D* were generated by PCR amplification from *Gossypium hirsutumt*. The amplified full-length fragments of *GhSPX1-1*, *GhSPX1-2*, *GhPHR1A*, *GhPHR1D*, *GhPHL1A,* and *GhPHL1D* were ligated into the pGADT7 and pGBKT7 vectors by homologous recombination. The constructs were confirmed by sequencing and transformed into Y2H Gold yeast cells. SD-Trp-Leu was used to select transformed positive clones, and SD-Leu-Trp-His-Ade was used to select positive interacting clones. The primers are listed in Table S1.

### 2.8. Protein Interaction Prediction

The AlphaFold 3.0 online (https://alphafoldserver.com/, accessed on 15 July 2025) website was used for predicting the structural model of two interacted proteins [43]. For each query pair, the amino acid sequences in FASTA format were submitted to the website. The predicted interface was evaluated by the model’s confidence metrics (pTM and ipTM scores).

### 2.9. RNA Extraction and qPCR

Total RNA of cotton fibers from different development stages was extracted via an RNA extraction kit (Tiangen, DP441, Beijing, China) with the standard method in the manual. Three-microgram samples of total RNA were used for first-strand complementary DNA (cDNA) synthesis using a RevertAid First Strand cDNA Synthesis Kit following the manufacturer’s instructions (Thermo, K1622, Thermo Fisher Scientific, Waltham, MA, USA). Quantitative real-time PCR (qRT-PCR) assays were performed using a Roche Light Cycle 480 II instrument (Roche, Basel, Switzerland). Three independent biological replicates were performed for each gene. The *GhUBQ7* gene (GenBank No. DQ116441.1) was used as internal reference gene. The 2^−ΔΔCT^ method was used to calculate the relative expression levels of each gene. The primers are listed in Table S1.

## 3. Results

### 3.1. Detection of G. hirsutum SPX Genes

The 20 SPX protein sequences of *Arabidopsis* were downloaded and utilized as query sequences. To determine the putative SPX protein in *G. hirsutum*, the SPX (PF03105) domain was confirmed by Pfam, CDD, and SMART. There were 44 *SPX* genes identified in the genome of *G. hirsutum*. Detailed information on all 44 SPX members, such as gene ID, gene name, protein length, molecular weight, predicted isoelectric point values, and estimated subcellular location, are presented in Table S2. A further analysis uncovered that the GhSPX proteins consisted of 184–831 amino acids, carrying a molecular weight of 21.7–95.8 kDa. An EXPASY analysis indicated that the SPX protein sequences had different isoelectric point (pI) values (range = 5.22–9.63). A subcellular location analysis showed that GhSPX proteins were localized in the cell membrane, chloroplast, nucleus, and vacuole. This characteristic implies that different subfamilies may exhibit varying biology functions.

### 3.2. Phylogenetic Analysis and Chromosomal Location of GhSPXs

**Figure 1:**
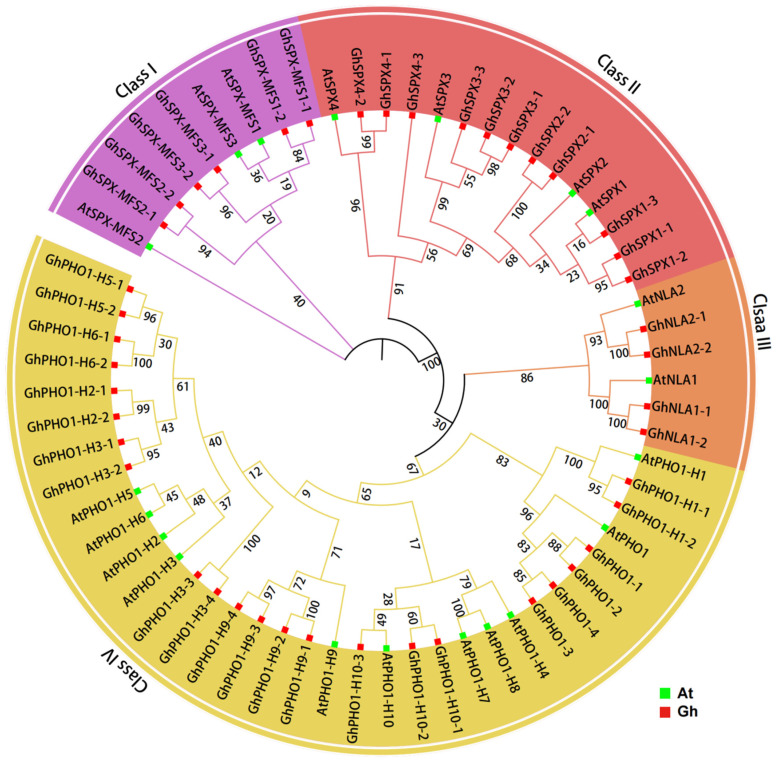
An unrooted phylogenetic tree of SPX protein sequences from *Arabidopsis thaliana* and *Gossypium hirsutum*. The phylogenetic tree was constructed by ClustalX 2.0 and MEGA7.0 software using the neighbor-joining (NJ) method and 1000 bootstrap replicates.

To illustrate the evolutionary relationship among SPX homologs in *Arabidopsis and G. hirsutum*, a phylogenetic tree was constructed comprising 44 *GhSPXs* in *G. hirsutum* and 20 *AtSPXs* in Arabidopsis. A total of 44 *GhSPXs* were named based on the corresponding Arabidopsis homologs. According to the phylogenetic tree, it clustered into four subclasses (Figure 1). Classes I, II, III, and IV contained 6, 11, 4, and 23 subfamily members, respectively. Class IV contained the most members, whereas class III contained the least. A phylogenetic analysis showed that SPX family members are highly conserved in different plants.

**Figure 2:**
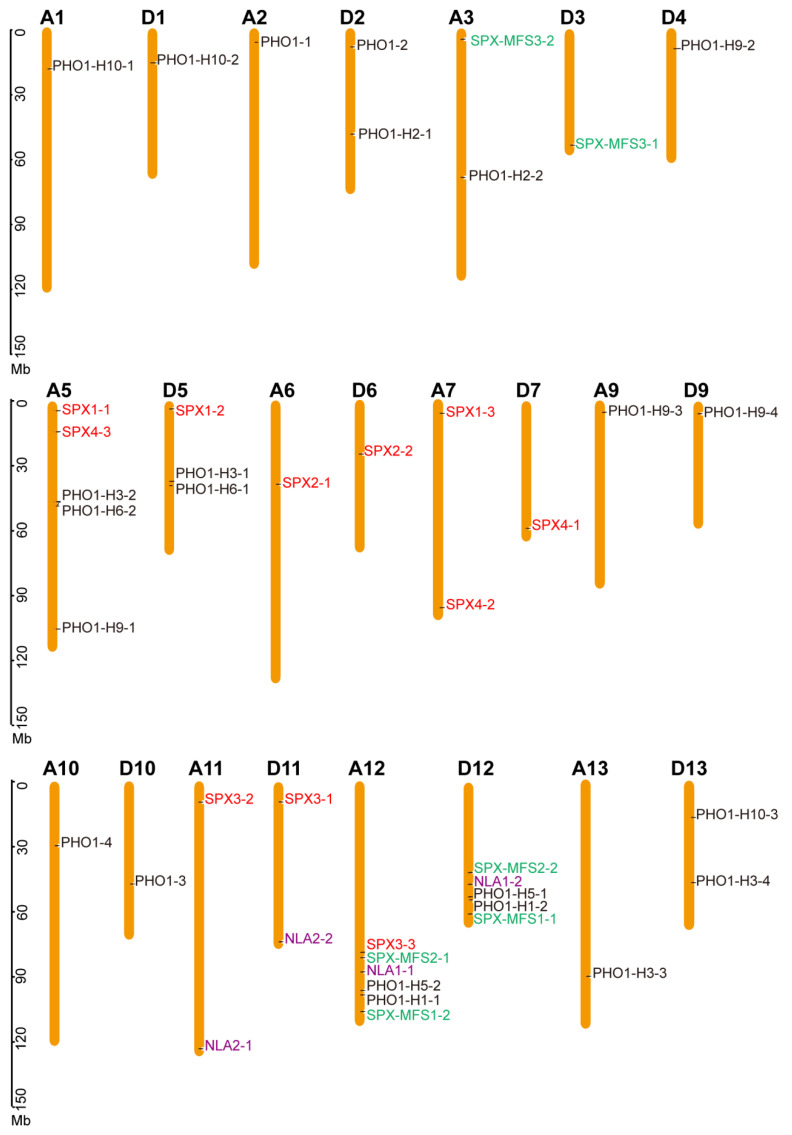
Chromosomal location of *GhSPX* genes on 23 chromosomes. The chromosome number is shown on the top of each chromosome. The scale bar indicates the length in megabases (Mb). The colors of *GhSPXs* means these *GhSPXs* from different subfamilies.

The chromosomal distribution of the 44 *GhSPX* genes is indicated in Figure 2. All *GhSPXs* identified could be mapped on 23 out of 26 *G. hirsutum* chromosomes, ranging from 1 to 6 per chromosomes, with Chr5 and Chr12 containing the most *GhSPX* genes. Further, *G. hirsutum* is an allotetraploid and consists of two different genomes, *viz*. AA and DD. The *G. hirsutum SPXs* also had two copies from chromosomes A and D (Figure 2). Some of the SPX and SPX-EXS (PHO1) subfamily genes lack a corresponding homolog. For example, *GhPHO1-H2-1* and *GhPHO1-H2-2*, localized to chromosome D2 and chromosome A3, respectively, lack homologs on chromosome A2 and chromosome D3. The results showed that the *GhSPX* genes are unevenly distributed among subgenomes A and D.

### 3.3. GhSPX Protein Domain and Gene Structure

**Figure 3:**
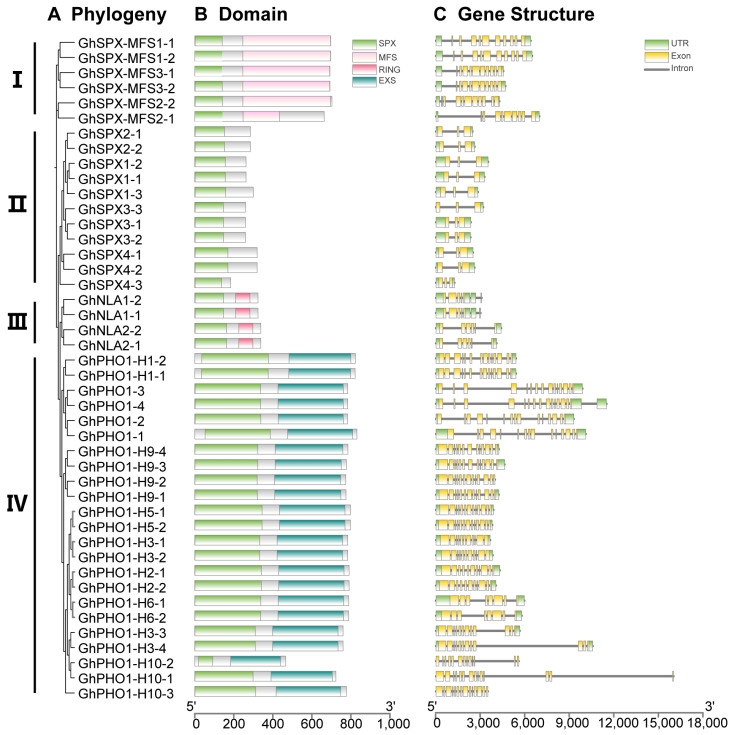
Phylogenetic relationships, domain, and gene structures of *GhSPX* genes in *Gossypium hirsutum*. (**A**) Phylogenetic tree of *GhSPX* genes in *Gossypium hirsutum*. (**B**) Domain analysis of *GhSPX* proteins in *Gossypium hirsutum*. The scale bar for protein length is indicated at the bottom. (**C**) Gene structure of *GhSPX* genes in *Gossypium hirsutum*. The scale bar for genomic length is indicated at the bottom.

In order to understand the DNA and protein sequence characteristics of cotton SPX family members, we analyzed their gene structure and protein domain. According to the phylogenetic tree, all GhSPX proteins were divided into four classes, including class I (SPX-MFS), class II (SPX), class III (NLA), and class IV (PHO1) (Figure 3A). Next, a protein domain analysis showed that all SPX proteins were divided into four classes according to C-terminal domain, including SPX, SPX-EXS, SPX-MFS, and SPX-RING (Figure 3B), consistent with the previous studies in Arabidopsis and wheat [36,44]. The structure of *GhSPX* genes was analyzed based on their UTR, exon, and intron. Most *GhSPXs* exhibited similar exon–intron organization. The SPX-MFS subfamily contains 10 exons, and SPX-RING contains 5–6 exons. SPX-EXS subfamily genes contain the largest number of exons (7–14), whereas SPX subfamily genes contain the fewest, only having two exons (Figure 3C). These results suggest that the SPX is the most conserved domain, and EXS is the most variable domain in cotton SPX family members.

### 3.4. Assessment of Cis-Acting Elements in GhSPX Promoters

**Figure 4:**
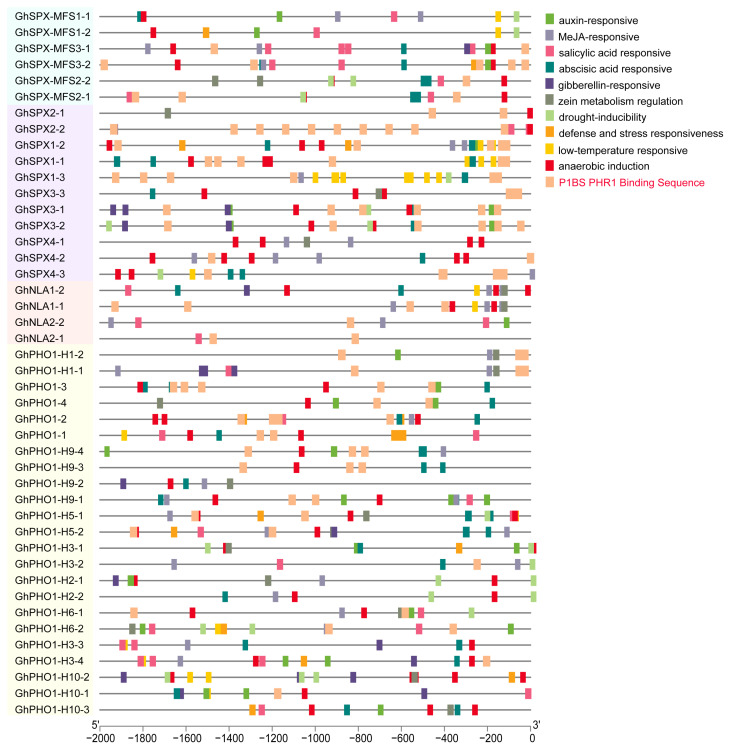
*Cis*-acting element analysis of SPX family members in *Gossypium hirsutum*. The colored boxes indicate different *cis*-elements in promoters of genes.

*Cis*-regulatory elements play important roles in the global regulation of gene expression, and genes with similar expression patterns may contain the same regulatory elements in their promoters. The promoters of 44 *GhSPX* genes were analyzed using the online software PlantCARE. Various types of *cis*-acting elements, including hormone response, stress response, low-temperature response, and PHR1 binding sequence elements, were detected in the promoter regions of *GhSPX* genes (Figure 4), and most *GhSPX* genes contained more than one *cis*-element type in their promoter regions, suggesting that these *GhSPX* genes may be involved in complex regulatory networks. The PHR1 binding sequence (P1BS), which contained an imperfect palindromic 8-bp sequence (GNATATNC) and was a conserved *cis*-element responding to Pi stress, was significantly enriched in the promoters of *GhSPXs* (Figure 4). The promoter region of SPX subfamily genes and SPX-EXS subfamily genes (includ *PHO1*, *PHO1-H1,* and *PHO1-H9*) has more P1BS *cis*-elements, and these P1BS *cis*-elements are closer to the start codon of SPX-EXS subfamily genes. These results suggest that these SPX-EXS subfamily genes may be regulated by GhPHR1 via GhPHR1 binding to P1BS *cis*-elements in promoter regions.

### 3.5. Expression Pattern of GhSPX Genes in Different Tissues and Different Abiotic Stress

**Figure 5:**
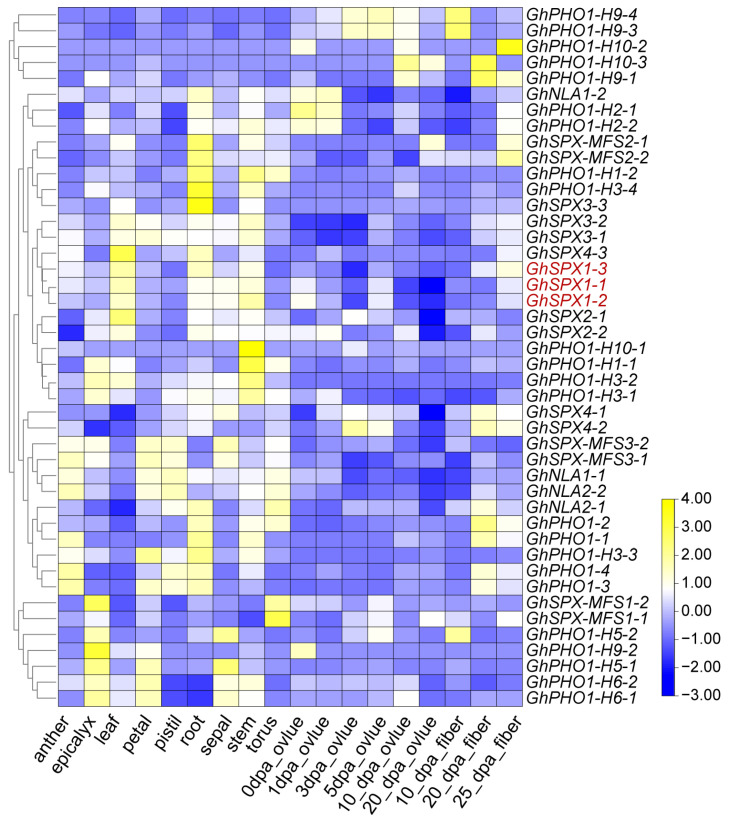
Expression profiles of *GhSPX* genes in different tissues and different cotton fiber development stages. Yellow and blue colors indicate low and high transcriptional expression levels, respectively. dpa, day post anthesis.

In order to further understand the gene function of *GhSPXs*, the expression pattern of *GhSPXs* in different tissues and under different abiotic stresses were analyzed. The expression of the GhSPX family member could be detected in different tissues and at different developmental stages, indicating that different SPX subfamily members participated in plant growth and development through various ways. Most GhSPX family members were expressed in the roots and stems, which was consistent with the function of SPX involved in P absorption and transport (Figure 5, Figure S1 and Table S3). In addition, under abiotic stress (high or low temperature, NaCl and PEG), some *GhSPX* genes (*PHO1*) were found to be significantly responsive to high (37 °C, 1 h) and low (4 °C, 24 h) temperatures, which is consistent with the inclusion of low-temperature response elements in the promoter region of SPX gene (Figure 4, Figure S2 and Table S3).

### 3.6. Analysis of GhPHR Expression Profiles and Promoter Cis-Acting Elements

**Figure 6:**
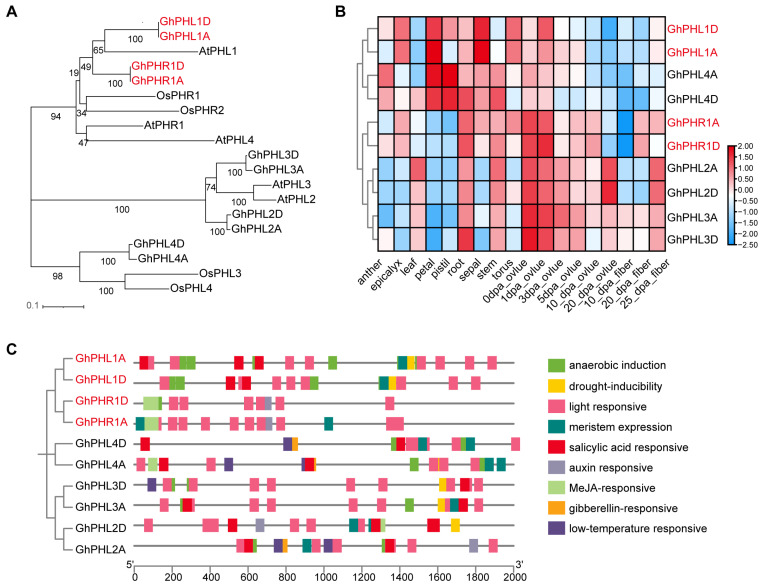
Basic information of some GhPHR genes in cotton. (**A**) Phylogenetic tree analysis of 5 *Arabidopsis thaliana PHR*-related genes, 4 rice *PHR*-related genes, and 10 cotton *PHR*-related genes. (**B**) Expression analysis of 10 *GhPHR*-related genes in different tissues and at different developmental stages. Red and blue colors indicate low and high transcriptional expression levels, respectively. dpa, day post anthesis. (**C**) Analysis of promoter *cis*-acting elements of 10 *GhPHR* related genes.

In Arabidopsis and rice, SPX can interact with PHR1 to prevent PHR1 binding to P1BS elements and then inhibit the expression of low phosphorus response genes [14,15]. In order to study PHR1 and PHL proteins in *G. hirsutum*, BlastP was used to search for proteins similar to Arabidopsis and rice PHR1 proteins in *G. hirsutum*. A total of 10 *GhPHR* and *GhPHL* genes in *G. hirsutum* were obtained (Figure 6A and Table S4). A further expression profiles analysis showed that a large number of *GhPHR* and *GhPHL* genes were expressed in the roots and stems (Figure 6B and Figure S3). In addition, the promoter of *GhPHR* and *GhPHL* genes in cotton contains a large number of light-responsive, salicylic acid-responsive, and Me JA-responsive elements (Figure 6C). It is suggested that the PHR gene may be involved in various biological processes besides low-phosphorus response.

### 3.7. Expression Profiles of GhSPXs and GhPHRs Under P Deficiency and P Supplement Conditions

**Figure 7:**
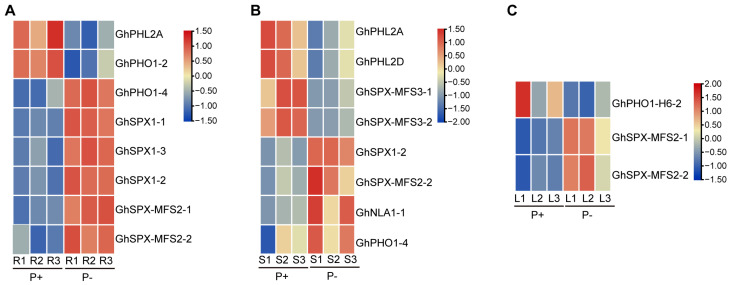
Expression levels of *GhPHR* and *GhSPX* family members in P abundance and P deficiency. (**A**) *GhPHR* and *GhSPX* family members differentially expressed in roots at different P concentrations. Red and blue colors indicate low and high transcriptional expression levels, respectively. P+, P abundance. P−, P deficiency. (**B**) *GhPHR* and *GhSPX* family members differentially expressed in stems at different P concentrations. (**C**) *GhPHR* and *GhSPX* family members differentially expressed in leaves at different P concentrations.

In order to explore the expression changes of *GhSPXs* and *GhPHRs* at different Pi concentrations, we used the published transcriptome data of cotton roots, stems, and leaves under Pi deficiency and Pi supplement conditions, and the corresponding 1679, 1611, and 841 differentially expressed genes for analysis [42]. A total of eight differentially expressed genes were detected in the roots, among which *GhPHL2A* and *GhPHO1-2* were up-regulated under Pi-sufficient conditions, while *GhPHO1-4*, *GhSPX1-1/1-2/1-3*, and *GhSPX-MFS2-1/2-2* were highly expressed under Pi deficiency conditions (Figure 7A). Also, eight differentially expressed genes were detected in the stems, among which *GhPHL2A/D* and *GHSPX-MFS3-1/2-2* were up-regulated under sufficient phosphorus conditions, and *GhSPX1-2*, *GhSPX-MFS2-2*, and *GhPHO1-4* were up-regulated under low-phosphorus conditions, similar to the roots. The expression level of *GhNAL1-1* in the stems was also down-regulated under low-phosphorus conditions (Figure 7B). The expression level of *GhPHO1-H6-2* in the leaves was up-regulated under P supplement conditions, and the expression level of *GhSPX-MFS2-1/2-1* was up-regulated under P-sufficient conditions, similar to that in the roots (Figure 7C). A total of 2 *GhPHL* genes and 11 *GhSPX* genes may be involved in the regulation of Pi response, and further functional studies are needed.

### 3.8. GhSPXs Interact with GhPHRs

**Figure 8:**
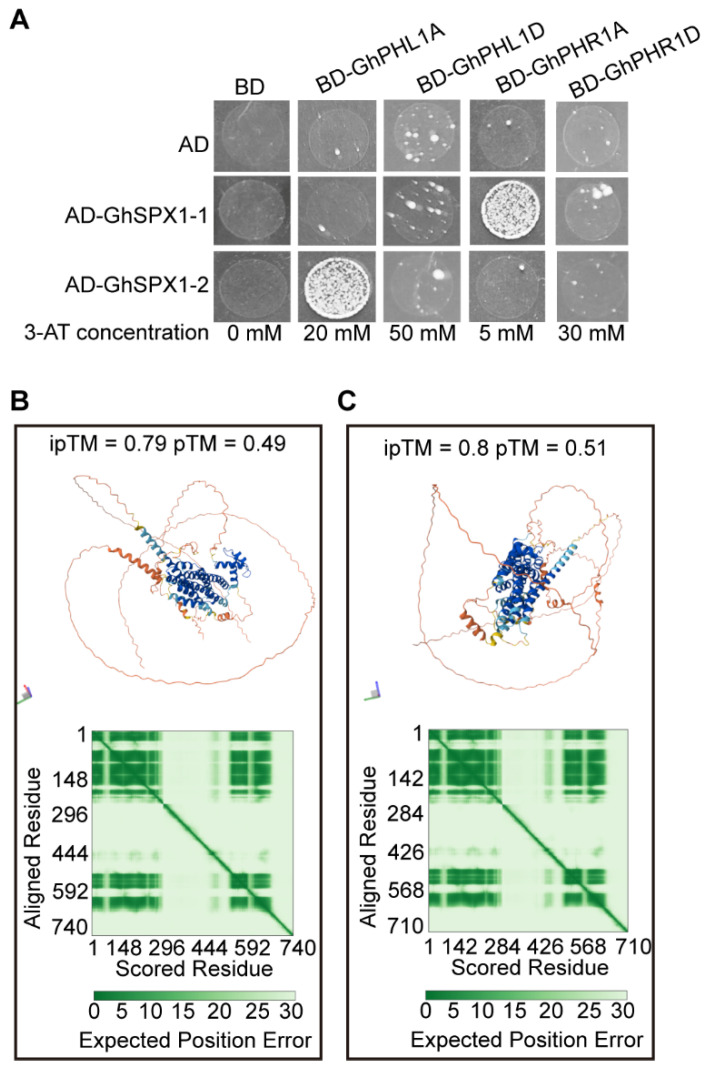
Interaction of GhSPX1-1, GhSPX1-2, and GhPHL1A, GhPHL1D, GhPHR1A, and GhPHR1D. (**A**) Yeast two-hybrid assay of two GhSPXs and four GhPHRs. (**B**) Structural model from the AlphaFold3.0 prediction of the interaction between full-length proteins of GhSPX1-1 and GhPHR1A. Heatmaps represent the Predicted Aligned Error (PAE) score between all pairs of residues. (**C**) Structural model from the AlphaFold3.0 prediction of the interaction between full-length proteins of GhSPX1-2 and GhPHL1A. Heatmaps represent the Predicted Aligned Error (PAE) score between all pairs of residues.

In Arabidopsis, the AtSPX1 shares the SPX domain with yeast Pi sensors, and the SPX1/PHR1 interaction is influenced by Pi directly [14]. To determine whether GhSPX1 interacts with GhPHR1 and GhPHL1 (PHR1-like 1), a Y2H assay was performed. First, we screened the 3AT concentrations that inhibited GhPHL1A-BD, GhPHL1D-BD, GhPHR1A-BD, and GhPHR1D-BD self-activation, which were 20 mM, 50 mM, 5 mM, and 30 mM, respectively. Then, GhSPX1-AD and GhPHR1-BD were co-transferred to yeast cells and SD-Trp-Leu-His-Ade selected media with different 3AT concentrations were used to verify the interaction. Only GhSPX1-1-AD/GhPHR1A-BD and GhSPX1-2-AD/GhPHL1A-BD can grow on the selected media (Figure 8A). A further interaction structural model of GhSPX1-1 and GhPHR1A, and GhSPX1-2 and GhPHL1A was predicted by AlphaFold3.0 to gain additional insight into the nature of these two interactions (Figure 8B,C). These results suggest that GhSPX1-1 interacts with GhPHR1A and GhSPX1-2 interacts with GhPHL1A.

## 4. Discussion

SPX family members have important functions in plant P absorption, transport, and homeostasis. This study systematically identified and characterized 44 SPX family genes in *Gossypium hirsutum*, providing valuable insights into their roles in phosphorus (P) signaling, uptake, and homeostasis.

The 44 GhSPX genes are unevenly distributed among subgenomes A and D, and some SPX genes are present in only one subgenome while lacking corresponding homologs in the other subgenome, with the potential reasons including: 1 subgenome-biased gene loss during polyploidization and subsequent diploidization processes; 2 differential evolutionary selection pressure acting on subgenomes A and D; 3 subfunctionalization or neofunctionalization leading to asymmetric retention of gene copies; and 4 incomplete genome assembly that may miss some homologous sequences. Further in-depth exploration is need to explain this phenomenon.

The findings highlight the conservation and diversity of SPX genes across plant species, with the SPX members in cotton divided into four classes according to protein domains, namely, SPX, SPX-EXS, SPX-MFS, and SPX-RING (Figure 1, Figure 2 and Figure 3). There are 20 and 15 SPX family members in Arabidopsis and rice, respectively, which are also divided into four classes [2], indicating an evolutionary conservation of SPX proteins in regulating P homeostasis. However, cotton exhibits a larger number of SPX genes (45) compared to Arabidopsis (20) and rice (15), likely due to its allotetraploid genome. The uneven distribution of these genes across subgenomes A and D, with some lacking homologs, hints at functional diversification or subfunctionalization post-polyploidization. Such an expansion may equip cotton with enhanced adaptability to P-deficient soils.

A *cis*-acting element analysis of SPX family members showed that most SPX family members had P1BS binding sites (Figure 4). In addition, seven SPX family members were detected to have up-regulated under P deficiency conditions (Figure 7), and the promoter region of these seven genes all contained P1BS binding sites. It is suggested that these seven genes may be regulated by *GhPHR1* in response to low-phosphorus stress. Previous studies have shown that phosphorus starvation can induce the expression of some SPX subfamily genes in Arabidopsis and rice [44,45], similar to cotton. The expression of three *GhSPX1* genes is up-regulated under low phosphorus stress, suggesting that *GhSPX1* may have similar functions to *SPX1* genes in Arabidopsis and rice.

The presence of P1BS *cis*-elements in the promoters of many GhSPX genes, particularly in the SPX and SPX-EXS subfamilies, suggests their regulation by PHR1, a central transcription factor in P signaling. The Y1H/EMSA is needed for validation in future work to test GhPHR1 binding to P1BS *cis*-elements in SPX-EXS subfamily gene promoters. The up-regulation of GhSPX1-1/1-2/1-3 and GhSPX-MFS2-1/2-2 under P deficiency indicates their involvement in P stress responses. Notably, GhSPX1-1 and GhSPX1-2 showed interactions with GhPHR1A and GhPHL1A, respectively, in yeast two-hybrid assays. These interactions likely form part of a negative feedback loop to fine-tune PHR1/PHL1 activity, ensuring balanced P uptake and utilization. The structural models predicted by AlphaFold3.0 further support the physical interactions between these proteins, providing a foundation for mechanistic studies (Figure S4).

Most GhSPX genes were highly expressed in roots and stems, consistent with their roles in P absorption and translocation. For instance, GhPHO1-4, a member of the SPX-EXS subfamily, was up-regulated under P deficiency, resembling AtPHO1 and OsPHO1;2, which mediate P transport from roots to shoots [23,46]. The differential expression of GhSPX genes in response to abiotic stresses, such as temperature extremes and salinity, implies their broader roles in stress adaptation. The enrichment of stress-responsive cis-elements in their promoters further supports this notion. These findings suggest that GhSPX genes integrate multiple environmental signals to modulate P homeostasis and stress responses.

In addition, previous studies have shown that SPX can inhibit the regulation of PHR1 on downstream low-phosphorus response genes through interaction with PHR1 [47]. We obtained a GhSPX1-1 interaction with GhPHR1A and a GhSPX1-2 interaction with GhPHL1A through a yeast two-hybrid experiment (Figure 8). It is suggested that GhSPX1-1 and GhSPX1-2 may negatively regulate the response of GhPHR1 and GhPHL1 to phosphorus starvation.

This study identified key GhSPX and GhPHR genes responsive to P deficiency, offering targets for improving P-use efficiency in cotton. For example, GhSPX-MFS2-1/2-2, homologous to vacuolar P transporters, may regulate P storage and remobilization, while GhSPX1-1/1-2 could modulate PHR1/PHL1 activity. Subsequently, transgenic function verification of these four genes can be carried out to further study their functions in cotton low-phosphorus response. Additionally, the interaction between GhSPX1-2 and GhPHL1A, a less-studied homolog of PHR1, warrants further investigation to uncover its unique regulatory functions.

## 5. Conclusions

In summary, 44 SPX genes were identified in the *G. hirsutum* genome, and their conserved domain, chromosomal distribution, and cis-acting elements were analyzed. The differential expression of SPX genes under P deficiency and P supplement conditions were analyzed using transcriptome data. A Y2H experiment screened two pairs of interacting proteins, GhSPX1-1/GhPHR1A and GhSPX1-2/GhPHL1A. Altogether, these findings present a reference basis for an enhanced understanding of the physiological roles of SPX genes in *G. hirsutum*.

## Data Availability

The original contributions presented in this study are included in the article/Supplementary Materials. Further inquiries can be directed to the corresponding author(s).

## Supplementary Materials

The following supporting information can be downloaded at https://www.mdpi.com/article/10.3390/biology14080916/s1, Figure S1: Expression analysis of *GhSPX1* in different cotton tissues; Figure S2: Expression analysis of *GhSPX* genes under different abiotic stress in *Gossypium hirsutum*; Figure S3: Expression analysis of *GhPHR1s* in different cotton tissues; Figure S4: Model of potential regulatory network involving GhPHRs and GhSPXs genes; Table S1: All primers used in this work; Table S2: Information of SPX gene family in *Gossypium hirsutum*; Table S3: Expression of 44 *GhSPX* Genes; Table S4: List of 10 PHR genes in *Gossypium hirsutum*.
