# Supplementary material for: GhSPX1s Interact with GhPHR1A and GhPHL1A in Regulating Phosphate Starvation Response in Cotton
Source: Biology (Basel). 2025 Jul 23;14(8):916. doi: 10.3390/biology14080916 (PMC12383507; doi:10.3390/biology14080916)
Supplement: Supplementary file 1 [file biology-14-00916-s001.zip › Figure S1-S4.pdf]

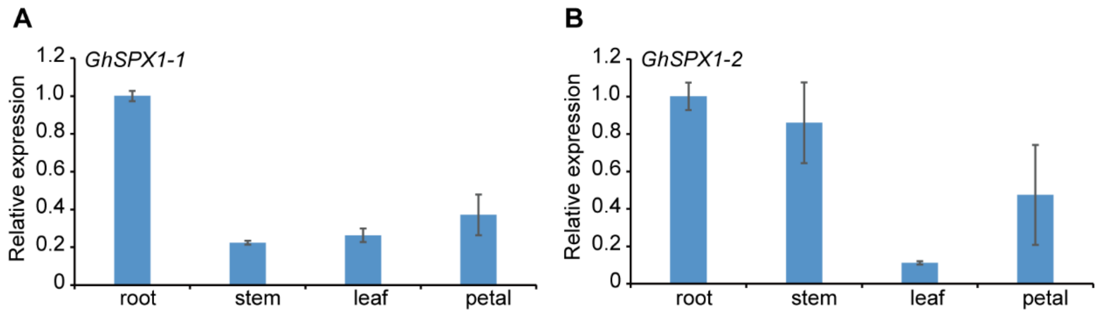

Figure S1. Expression analysis of *GhSPX1* in different cotton tissues.

Relative expression level of *GhSPX1-1* (A) and *GhSPX1-2* (B) in different cotton tissues. Error bars represent ± SD (n = 3).

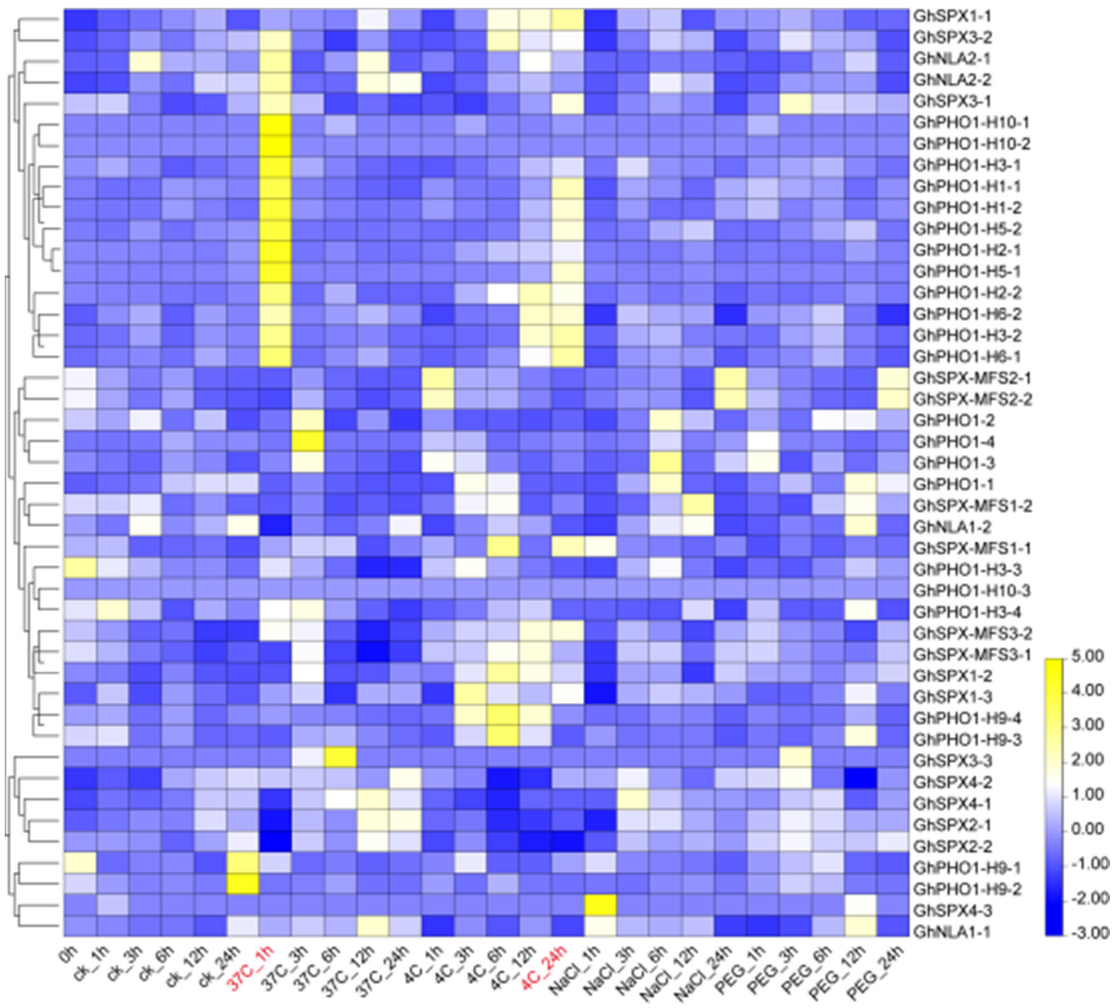

Figure S2. Expression analysis of *GhSPX* genes under different abiotic stress in *Gossypium hirsutum*.

Yellow and blue colors indicate low and high transcriptional expression levels, respectively. ck, control. h, hour.

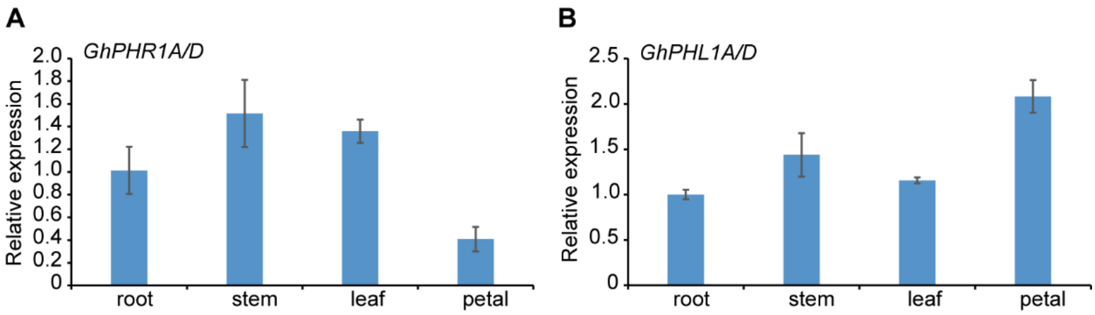

Figure S3. Expression analysis of *GhPHR1s* in different cotton tissues.

Relative expression level of *GhPHR1A/D* (A) and *GhPHL1A/D* (B) in different cotton tissues. Error bars represent ± SD (n = 3).

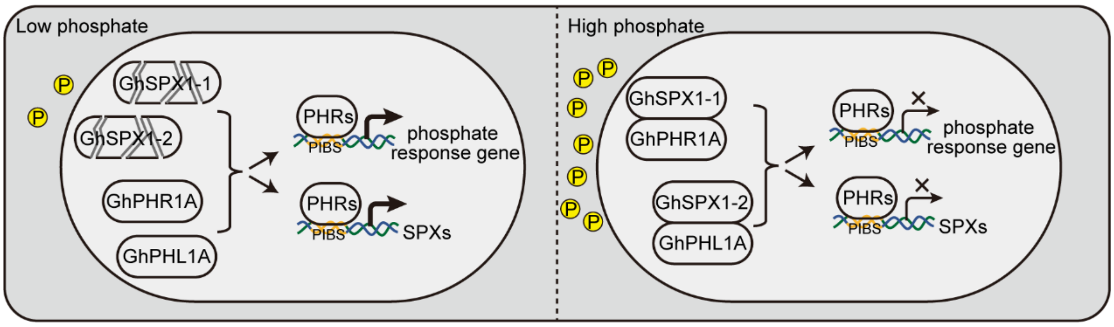

Figure S4. Model of potential regulatory network involving GhPHRs and GhSPXs genes.
