# Supplementary material for: GhSPX1s Interact with GhPHR1A and GhPHL1A in Regulating Phosphate Starvation Response in Cotton
Source: Biology (Basel). 2025 Jul 23;14(8):916. doi: 10.3390/biology14080916 (PMC12383507; doi:10.3390/biology14080916)
Supplement: Supplementary file 1 [file biology-14-00916-s001.zip › Table S1.pdf]

Table S1 All primers used in this work.

Primer	Sequence (5'-3')
AD-GhSPX1-1-EcoRI-F	gaggccagtgaattcATGAAATTCGGGAAGAGCCTT
AD-GhSPX1-1-BamHI-R	gagctcgatggatccCTACTTGGCTGCTTGTTCGAG
AD-GhSPX1-2-EcoRI-F	gaggccagtgaattcATGAAATTTGGAAAGAGCCTTAGTAGCCAG
AD-GhSPX1-2-BamHI-R	gagctcgatggatccCTATTTGGCTGCTTGTTCAAGAACAGGGA
BD-GhPHL1D-EcoRI-F	tggccatggaggccgaattcATGCCTTCATCTTTTCTGGCTC
BD-GhPHL1D-SalI-R	tgcggccgctgcaggtcgacCTATTTCTCTGTTCTTGCACGTTTTG
BD-GhPHR1A-EcoRI-F	gccatggaggccgaattcATGGAGGCACGACCTGCTTTATCC
BD-GhPHR1A-SalI-R	cggccgctgcaggtcgacTTATTCTTCGATTCGTGAACGCTTTGCG
BD-GhPHL1A-EcoRI-F	tggccatggaggccgaattcATGGAGAATTCTGTATCGCTTCAAG
BD-GhPHL1A-SalI-R	tgcggccgctgcaggtcgacCTATTTCTCTGTTCTTGCACGTTTTG
BD-GhPHR1D-EcoRI-F	tggccatggaggccgaattcATGGAGGCACGACCTGCTTTA
BD-GhPHR1D-SalI-R	tgcggccgctgcaggtcgacTTATTCTTCGATTCGTGAACGCT
GhSPX1-1-qrt-F	GGACTTCCATGGCGAGATGGTT
GhSPX1-1-qrt-R	AGCGGATCAATGCACCAGTTCT
GhSPX1-2-qrt-F	TGTTGCCGGTGCCGTAGATAAG
GhSPX1-2-qrt-R	TGCCTTTGCCACACTGTCTTGT
GhPHR1A/D-qrt-F	GACGACTAGGCCTCAGGTCCAT
GhPHR1A/D -qrt-R	AGTGATGCAGTATCAGCTGGCG
GhPHL1D/D -qrt-F	GAACGGCCAGATACATGCCAGA
GhPHL1D/D -qrt-R	CCTGCAATCGCAGTGCTTCAGT
GhUBQ-F	CCGCATTAGGGCACTCTTTTC
GhUBQ-R	GGCATTCCACCTGACCAACAA
