# Supplementary material for: GhSPX1s Interact with GhPHR1A and GhPHL1A in Regulating Phosphate Starvation Response in Cotton
Source: Biology (Basel). 2025 Jul 23;14(8):916. doi: 10.3390/biology14080916 (PMC12383507; doi:10.3390/biology14080916)
Supplement: Supplementary file 1 [file biology-14-00916-s001.zip › Table S2.pdf]

Table S2 Information of SPX gene family in *Gossypium hirsutum*.

Gene name	Gene ID	Protein length (aa)	MW (Da)	pI	Subcellular location
GhNLA1-1	GhM_A12G1701	324	37096.36	8.88	Nucleus.
GhNLA1-2	GhM_D12G1650	324	37024.2	8.68	Nucleus.
GhNLA2-1	GhM_A11G3957	339	39046.29	8.47	Nucleus.
GhNLA2-2	GhM_D11G3864	339	39093.41	8.57	Nucleus.
GhPHO1-1	GhM_A02G0457	831	95800.95	9.25	Cell membrane. Vacuole.
GhPHO1-2	GhM_D02G0450	783	90317.63	9.41	Cell membrane. Vacuole.
GhPHO1-3	GhM_D10G1833	784	90564	9.44	Cell membrane. Chloroplast. Vacuole
GhPHO1-4	GhM_A10G1282	784	90491.93	9.43	Cell membrane. Chloroplast. Vacuole
GhPHO1-H10-1	GhM_A01G1049	723	84206.37	9.63	Cell membrane.
GhPHO1-H10-2	GhM_D01G1013	466	54518.11	9.46	Cell membrane.
GhPHO1-H10-3	GhM_D13G0882	777	89921.46	8.79	Cell membrane. Vacuole.
GhPHO1-H1-1	GhM_A12G2200	822	95656.93	9.17	Cell membrane. Vacuole.
GhPHO1-H1-2	GhM_D12G2111	823	95711.03	9.07	Cell membrane. Vacuole.
GhPHO1-H2-1	GhM_D02G1497	791	91356.9	9.32	Cell membrane. Vacuole.
GhPHO1-H2-2	GhM_A03G1373	791	91462.03	9.37	Cell membrane. Vacuole.
GhPHO1-H3-1	GhM_D05G3472	784	91001.41	9.39	Cell membrane. Vacuole.
GhPHO1-H3-2	GhM_A05G3524	784	90973.29	9.39	Cell membrane.
GhPHO1-H3-3	GhM_A13G1681	760	88259.96	9.37	Cell membrane.
GhPHO1-H3-4	GhM_D13G1558	760	88569.22	9.27	Chloroplast. Vacuole.
GhPHO1-H5-1	GhM_D12G2012	798	92647.88	9.31	Chloroplast. Vacuole.
GhPHO1-H5-2	GhM_A12G2110	798	92600.85	9.28	Cell membrane.
GhPHO1-H6-1	GhM_D05G3473	789	91521.42	9.37	Chloroplast. Vacuole
GhPHO1-H6-2	GhM_A05G3527	788	91544.5	9.35	Chloroplast. Vacuole.
GhPHO1-H9-1	GhM_A05G4401	775	89279.69	9.4	Cell membrane. Vacuole.
GhPHO1-H9-2	GhM_D04G0542	774	89048.27	9.37	Cell membrane. Vacuole.
GhPHO1-H9-3	GhM_A09G0158	777	89769.49	9.47	Nucleus. Vacuole.
GhPHO1-H9-4	GhM_D09G0157	785	90637.41	9.4	Cell membrane. Vacuole.
GhSPX1-1	GhM_A05G0175	263	30328.84	6.28	Nucleus. Vacuole.
GhSPX1-3	GhM_A07G0453	300	34174.13	5.22	Nucleus. Vacuole.
GhSPX1-2	GhM_D05G0185	263	30355.91	6.8	Vacuole.
GhSPX2-1	GhM_A06G1194	285	32981.86	5.43	Vacuole.
GhSPX2-2	GhM_D06G1222	285	32838.74	5.54	Nucleus. Vacuole.
GhSPX3-1	GhM_D11G0926	260	30170.74	5.71	Vacuole.
GhSPX3-2	GhM_A11G0911	260	30170.78	5.86	Vacuole.
GhSPX3-3	GhM_A12G1272	261	30344.35	7.69	Chloroplast. Vacuole.
GhSPX4-1	GhM_D07G2561	320	36236.78	5.41	Nucleus. Vacuole.
GhSPX4-2	GhM_A07G2660	320	36372.98	5.42	Nucleus. Vacuole.
GhSPX4-3	GhM_A05G1393	184	21672.26	9.28	Nucleus. Vacuole.
GhSPX-MFS1-1	GhM_D12G2689	697	78151.78	6.62	Cell membrane. Vacuole
GhSPX-MFS1-2	GhM_A12G2801	697	78179.79	6.45	Cell membrane. Vacuole.
GhSPX-MFS2-1	GhM_A12G1372	663	74620.88	6.71	Vacuole.
GhSPX-MFS2-2	GhM_D12G1314	703	78657.34	8.3	Vacuole.
GhSPX-MFS3-1	GhM_D03G1869	693	77665.03	5.86	Vacuole.
GhSPX-MFS3-2	GhM_A03G0245	693	77792.21	6.08	Vacuole.
