# Supplementary material for: GhSPX1s Interact with GhPHR1A and GhPHL1A in Regulating Phosphate Starvation Response in Cotton
Source: Biology (Basel). 2025 Jul 23;14(8):916. doi: 10.3390/biology14080916 (PMC12383507; doi:10.3390/biology14080916)
Supplement: Supplementary file 1 [file biology-14-00916-s001.zip › Table S4.pdf]

Table S4 List of 10 PHR genes in *Gossypium hirsutum*

Gene name	Gene ID
GhPHR1A	GhM_A11G1564
GhPHR1D	GhM_D11G1558
GhPHL1A	GhM_A11G3088
GhPHL1D	GhM_D11G3020
GhPHL3A	GhM_A10G1517
GhPHL3D	GhM_D10G1602
GhPHL4A	GhM_A10G0026
GhPHL4D	GhM_D10G0017
GhPHL2A	GhM_A02G0277
GhPHL2D	GhM_D02G0300
